# Characterization of limbal explant sites: Optimization of stem cell outgrowth in *in vitro* culture

**DOI:** 10.1371/journal.pone.0233075

**Published:** 2020-05-14

**Authors:** Pattama Ekpo, Naharuthai Inthasin, Sutthicha Matamnan, Patimaporn Wongprompitak, Methichit Wattanapanitch, Chawikan Boonwong, Chareenun Chirapapaisan, Panotsom Ngowyutagon, Mongkol Uiprasertkul, Pinnita Prabhasawat, Wiwit Tantibhedhyangkul

**Affiliations:** 1 Department of Immunology, Faculty of Medicine Siriraj Hospital, Mahidol University, Bangkok, Thailand; 2 Research Department, Faculty of Medicine Siriraj Hospital, Mahidol University, Bangkok, Thailand; 3 Research Department, Siriraj Center for Regenerative Medicine (SiCRM), Faculty of Medicine Siriraj Hospital, Mahidol University, Bangkok, Thailand; 4 Department of Ophthalmology, Faculty of Medicine Siriraj Hospital, Mahidol University, Bangkok, Thailand; 5 Department of Pathology, Faculty of Medicine Siriraj Hospital, Mahidol University, Bangkok, Thailand; Cedars-Sinai Medical Center, UNITED STATES

## Abstract

Simple limbal epithelial transplantation (SLET) and cultivated limbal epithelial transplantation (CLET) are proven techniques for treating limbal stem cell deficiency (LSCD). However, the precise regions that are most suitable for preparing explants for transplantation have not been identified conclusively. Accordingly, this *in vitro* study aimed at determining ideal sites to be selected for tissue harvest for limbal stem cell culture and transplantation. We evaluated cell outgrowth potential and the expression of stem cell markers in cultures from 48 limbal explants from five cadaveric donors. The limbal explants were generated from the three specific sites: Lcor (located innermost and adjacent to the cornea), Lm (middle limbus), and Lconj (located outermost adjacent to the conjunctiva). We found that explants from the Lconj and Lm sites exhibited higher growth potential than those from the Lcor site. Transcript encoding the stem cell marker and p63 isoform, ΔNp63, was detected in cells from Lm and Lconj explants; expression levels were slightly, though significantly (*p*-value < 0.05), higher in Lm than in Lconj, although expression of ΔNp63α protein was similar in cells from all explants. Differential expression of ATP-Binding Cassette Subfamily G Member 2 (*ABCG2*) did not reach statistical significance. Immunohistochemistry by indirect immunofluorescence analysis of limbus tissue revealed that the basal layer in explant tissue from Lconj and Lm contained markedly more stem cells than found in Lcor explant tissue; these findings correlate with a higher capacity for growth. Collectively, our findings suggest that explants from the Lconj and Lm sites should be selected for limbal cell expansion for both CLET and SLET procedures. These new insights may guide surgeons toward specific limbal sites that are most suitable for stem cell culture and transplantation and may ultimately improve treatment outcomes in the patients with LSCD.

## Introduction

Limbal epithelial stem cells reside in the limbus, which is the area of the eye located directly between the cornea and conjunctiva. These stem cells have the capacity for self-renewal, are capable of proliferation and migration, and are indispensable for continuous regeneration of the corneal epithelium [1–4]. Limbal stem cell deficiency (LSCD) is a clinical condition resulting from limbal epithelial stem cell damage. LSCD can result from conditions including aniridia, Stevens-Johnson syndrome, contact lens wear, and thermal and alkali injuries [5]. Damage to the limbal stem cell population results in impaired cell turnover, corneal conjunctivalization, and neovascularization (i.e., invasion of the cornea by the conjunctival epitheliums and blood vessels) [6].

Current treatments for LSCD that aim to restore the defective corneal stem cell population include cultivated limbal epithelial transplantation (CLET) [7, 8], cultivated oral mucosal epithelial transplantation [9, 10], and simple limbal epithelial transplantation (SLET) [11, 12]. CLET and SLET utilize limbal explant tissue from donor eyes to repair a recipient’s damaged cornea [13–15]. Typically, the amount of tissue removed is minimized to prevent damage to the donor eye; however, a sufficient number of limbal stem cells that can survive and proliferate are required for a successful procedure. As ocular structure varies among individuals, it may be difficult for an ophthalmic surgeon to determine which region of the limbus might be ideal for effective transplantation. As such, additional information about stemness and proliferation potential of various sites within the limbus would serve to improve harvesting techniques.

Several groups have described the ideal size of a limbal explant that will yield the greatest degree of stemness in cell cultures [16–19]. In a recent study, stemness of explants from four different locations within the limbus, including superior, inferior, nasal, and temporal, were compared; the results indicated that the superior and inferior limbal areas exhibited greater growth potential [20]. However, to our knowledge, there have been no studies that aim to identify stemness and growth potential of cells from the limbus with respect to the proximity to the cornea and conjunctiva. Accordingly, in this study, we asked whether limbal stem cells from three different areas (innermost and adjacent to the cornea, outermost and adjacent to the conjunctiva, or at the midpoint) exhibit differential growth and expression of stem cell markers in *in vitro* culture.

In this study, we aim to explore cell outgrowth and expression of stem cell markers in cells from explants from three sites within the limbus, which we have identified as Lcor (innermost and adjacent to the cornea), Lm (middle limbus), and Lconj (outermost and adjacent to the conjunctiva). We also identified and quantified stem cells in explants and in outgrowth cells from each of these three sites. An improved understanding of differential cell growth and stemness of cells from explants can be used to direct clinical stem cell transplantation and may result in improved treatment outcomes for CLET and SLET.

## Materials and methods

### Limbal tissue

**Fig 1:**
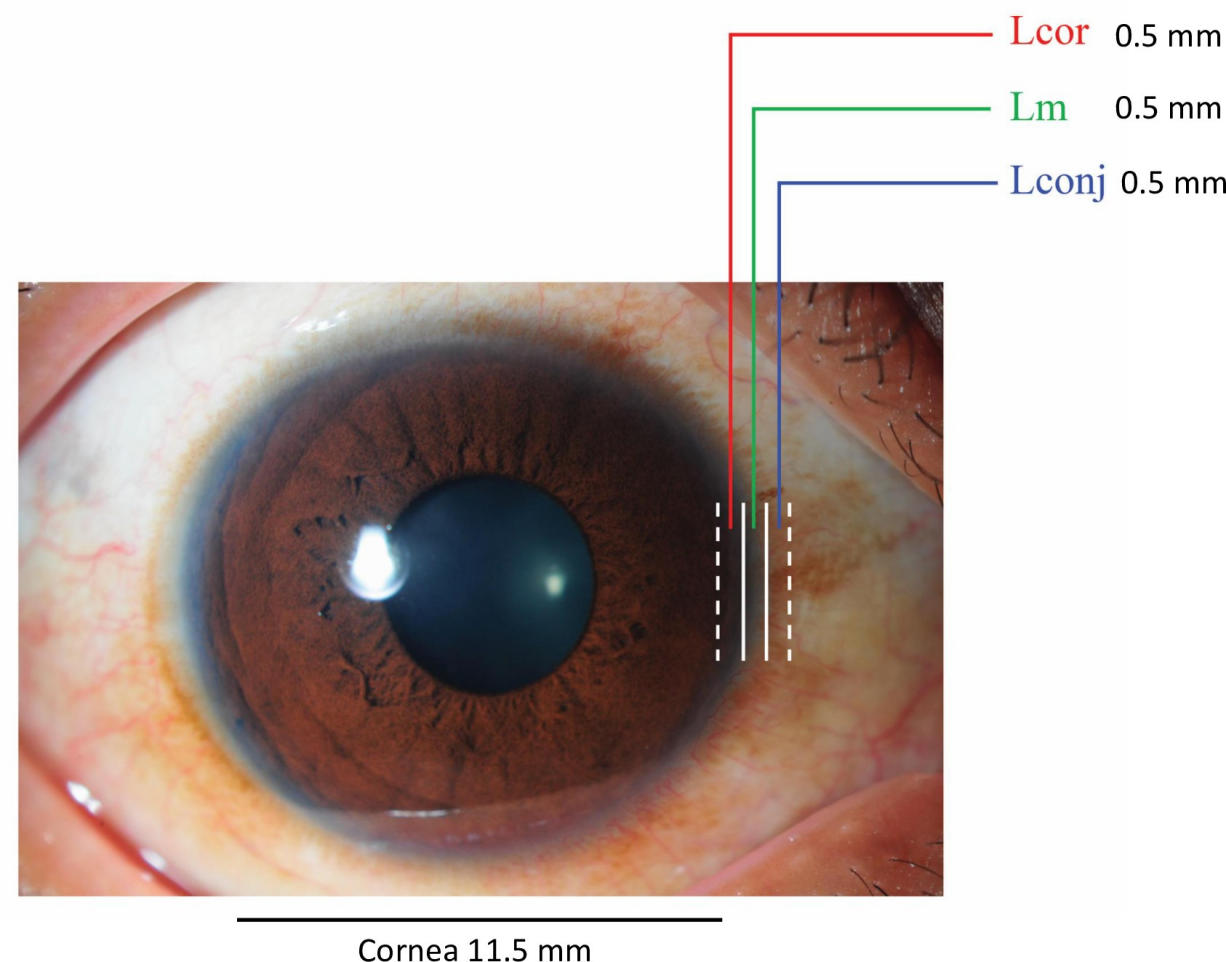
Demarcation of three sites within the limbus. Lcor, located innermost and adjacent to the cornea; Lm, middle of the limbus; Lconj, located outermost and adjacent to the conjunctiva.

Limbal tissue was obtained from five cadaveric donors provided by the Thai Red Cross Society. The study protocol was approved by Siriraj Institutional Review Board of the Faculty of Medicine Siriraj Hospital, Mahidol University, Thailand (protocol number: si709/2016). The mean age of donors was 51.2 years (range: 37–61). We preserved five corneoscleral tissues in hypothermic eye bank storage conditions (4°C) for 2–5 days before sample preparation. Limbal preparation was performed under the ophthalmic surgical microscope Proveo 8 (Leica Microsystems Inc., Buffalo Grove, IL, USA). The 12-o’clock position in corneoscleral rim was not specified. Each limbal ring was cut into five smaller sections of an approximate size of 1.5 × 3.0 mm. One of the five pieces from each ring was further dissected into subsections that include Lcor, Lm, and Lconj regions as defined above (Fig 1). Each subsection had an approximate size of 0.5 × 3.0 mm. Superficial tissues from Lconj, Lm, and Lcor subsections were used for cultivation. Overall, we selected 16 limbal tissues and divided into 48 individual subsections (16 sets of Lcor, Lm, and Lconj), which were used for cultivation. The remaining 9 sets of full-thickness limbal tissue were embedded in the optimal cutting temperature compound (Tissue-Tek, Torrance, CA). Frozen tissue was cryo-sectioned at a thickness of 7 μm and then stained with hematoxylin and eosin (H&E) or analyzed by immunohistochemistry (IHC) using indirect immunofluorescence methods.

### Cultivation of human limbal explants

Human limbal explant culture was performed as previously described [7]. Briefly, superficial limbal tissues from Lconj, Lm, and Lcor were washed three times in phosphate-buffered saline (PBS) and then incubated in dispase for 20 minutes at 37°C. After three additional washes with PBS, the limbal explants were placed in a 24-well tissue culture plate with the epithelium facing up. They were then submerged in CELLnTEC-Prime^®^ (CnT-Prime) medium supplemented with amino acids, minerals, vitamins, organic compounds, transferrin, insulin, epithelial growth factor, and fibroblast growth factor (CELLnTEC, Bern, Switzerland) and 10 μM Y27632, a Rho-associated protein kinase (ROCK) inhibitor (FUJIFILM Wako Pure Chemical Corp, Osaka, Japan). The medium was replaced every two days. Outgrowth from the limbal explants was recorded, and expression of stem cell markers in confluent limbal cell cultures was evaluated by indirect IHC and quantitative reverse transcription-polymerase chain reaction (qRT-PCR).

### Immunocytochemistry and immunohistochemistry

Cultured cells and tissue samples were fixed with 4% paraformaldehyde for 10 minutes and washed three times with PBS for 5 minutes prior to permeabilization with 0.1% Triton X-100 (Sigma-Aldrich Corporation, St. Louis, MO, USA) for 10 minutes. The samples were washed and blocked with 2.5% bovine serum albumin (BSA) in PBS (BSA-PBS) for 30 minutes at room temperature (RT). After washing, the samples were incubated with primary antibodies, including mouse monoclonal anti-human ΔNp63 (clone BC28, catalog number ab172731, diluted 1:50 in 0.1% BSA-PBS; Abcam, Cambridge, UK), and rabbit polyclonal anti-human p63α (catalog number 4892, diluted 1:100 in 0.1% BSA-PBS; Cell Signaling Technology, Danvers, MA, USA), mouse monoclonal anti-human p63 primary antibody (clone 4A4, catalog number ab735, diluted 1:50 in 0.1% BSA-PBS; Abcam), or their isotype-control antibodies at the same concentrations (Abcam) at 4°C overnight. The samples were then washed and incubated with secondary antibodies, including Alexa Fluor 568-conjugated goat anti-mouse IgG and Alexa Fluor 488-conjugated goat anti-rabbit IgG (both diluted 1:200 in 0.1% BSA-PBS; Invitrogen, Carlsbad, CA, USA) at RT for one hour. After washing, 300 nM 4′, 6-diamidino-2-phenylindole (DAPI) (Sigma-Aldrich) was used for nuclear staining. The slides were mounted with Mowiol^®^ 40–88 (Sigma-Aldrich). The stained cells were observed at 200× magnification on an inverted fluorescent microscope (Nikon Instruments Inc., Melville, NY). The ΔNp63α -positive cells were identified by the colocalization of red (p63α) and green (ΔNp63) fluorescent signals in the cell nuclei. More than 1000 cells were scored for each condition (NIS Element software, Nikon Instruments). The percent positive cells were calculated from the number of fluorescent cells divided by total cells scored (x100). Positive cells in frozen tissue samples were identified with anti-p63 antibody (clone 4A4) as red fluorescent signals in the cell nuclei.

### Quantitative reverse transcription-polymerase chain reaction (qRT-PCR)

Total RNA from limbal cells in culture was extracted using the GenUP Total RNA Kit (Biotechrabbit, Hennigsdorf, Germany) following the manufacturer’s protocol. cDNA was synthesized by Superscript^TM^ III First-Strand Synthesis System (Invitrogen), and qRT-PCR was performed using iTaq SYBR Green Master Mix (Bio-Rad Laboratories, Hercules, CA, USA). The primer sequences used in this study are listed in Table 1.

**Table 1:** Primer sequences used for qRT-PCR.

Gene	Sequence (5’->3’)	Product size (bp)
Δ*Np63_F*	GGAAAACAATGCCCAGACTC	125
Δ*Np63_R*	GCGCGTGGTCTGTGTTATAG	
*ABCG2_F*	GAGCCTACAACTGGCTTAGACTCAA	85
*ABCG2_R*	TGATTGTTCGTCCCTGCTTAGAC	
*GAPDH_F*	ACAGCCTCAAGATCATCAGCA	119
*GAPDH_R*	GATGGCATGGACTGTGGTCA	

**Abbreviation:** bp, base pair

References [21–23]

The qRT-PCR amplification of target cDNAs included the following steps: initial activation at 95°C for 1 minute, denaturation at 95°C for 10 seconds, annealing at 58°C for 15 seconds, and extension at 72°C for 5 seconds. The process was repeated for 45 cycles. Quantification cycles (Cq) of the target gene and the housekeeping gene, *GAPDH*, were used to calculate the fold change using the following formula: FC = 2^-ΔCq^, ΔCq = (Cq of target gene–Cq of *GAPDH*).

### Statistical analysis

IBM SPSS Statistics v24 was used for statistical analysis. Data in each experiment were presented as mean ± standard error of mean (SEM). Data from different groups were compared using Chi-square, paired t-test, or ANOVA, as indicated in each figure legend. A *p*-value < 0.05 was considered significant.

## Results

### Growth of limbal explants from three distinct sites in *in vitro* culture

The sites we have identified as Lconj, Lm, and Lcor within the circumcorneal transitional zone between the clear cornea on one side and the opaque sclera on the opposite side are shown in Fig 1.

**Fig 2:**
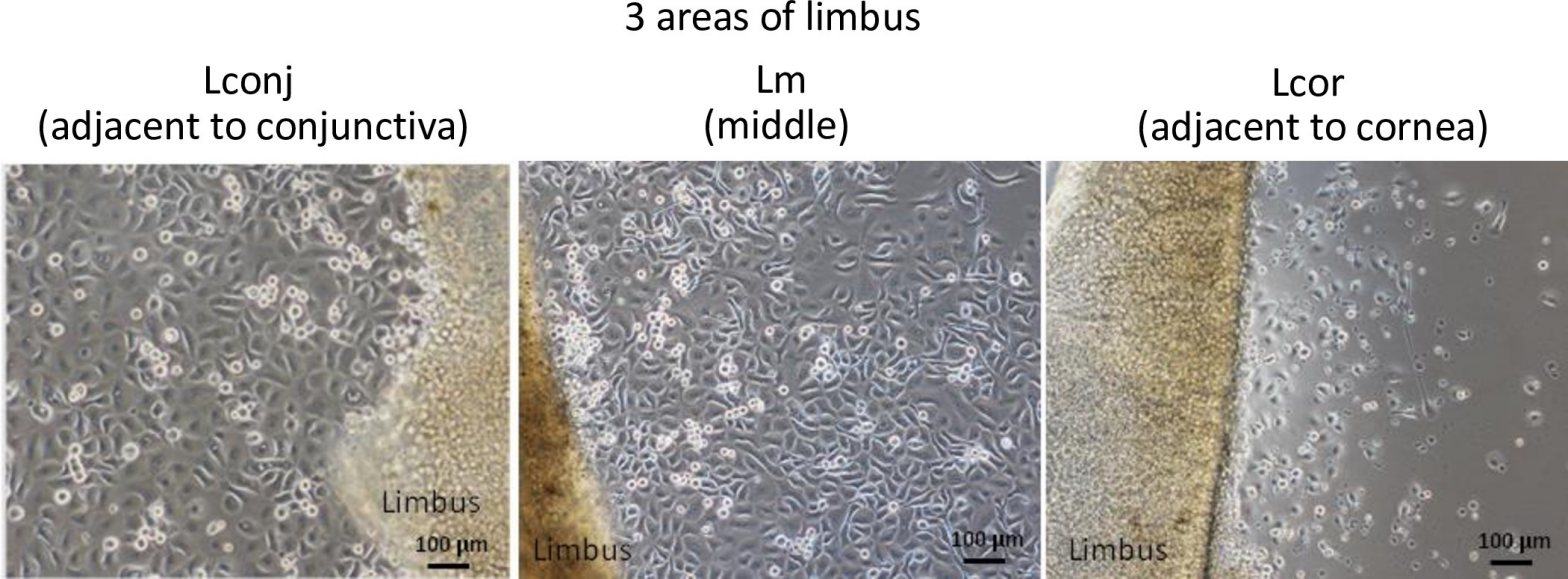
Limbal explant cultures. Representative limbal explant cultures from Lconj, Lm, and Lcor at day 7. All outgrowths included small cuboidal cells with high nucleus-to-cytoplasm ratios (scale bar = 100 μm).

Cultured cells from explant outgrowths were maintained in the CnT-Prime medium until they reached 70%–80% confluence (at days 7–14; Fig 2). Successful explant expansion was characterized by outgrowth of cells with cuboidal-like morphology and a diameter of >5 mm within 14 days (Fig 5). Ten out of 16 Lconj explants and 13 out of 16 Lm explants achieved satisfactory expansion, whereas only 5 of 16 Lcor explants grew successfully (Lm > Lcor outgrowth, *p* < 0.01). The cell outgrowth of explants from Lconj was somewhat higher than that of Lcor, although the difference did not reach statistical significance, possibly due to the limited number of tissue samples evaluated (Table 2).

**Table 2:** Limbal tissue and cell outgrowth data.

Donors	Number of explants that demonstrated cell outgrowth			Days of cultivation to confluence^#^
	Lconj	Lm	Lcor	
1	3/3	3/3	0/3	14
2	0/3	3/3	1/3	11
3	3/3	2/3	1/3	10
4	3/3	2/3	1/3	7
5	1/4	3/4	2/4	11
Total explant outgrowth	10/16	13/16	5/16	
Percent of success (95% CI)	62.5% (38.6–81.5%)	81.3%* (57–93.4%)	31.3%* (14.2–55.6%)	

Abbreviations: Lconj, limbus adjacent to the conjunctiva; Lm, middle limbus; and Lcor, limbus adjacent to the cornea.

A successful culture was defined as one in which the diameter of cell outgrowth was larger than 5 mm within 14 days

*p < 0.01 Lm *vs*. Lcor, by Chi-square test, 95% confidence interval (95% CI).

^#^The duration that the explants from each donor reach confluence monolayers. This parameter was similar in all expandable explants froms 3 limbal sites of the same donor but varied among different donors.

Interestingly, we discovered that animal-component-free CnT-Prime^®^ medium required supplementation with the ROCK inhibitor (Y27632); limbal explants were unable to sustain adequate growth in the absence of this inhibitor (S1 Fig).

### Detection of stem cell markers in corneal limbal outgrowths

**Fig 3:**
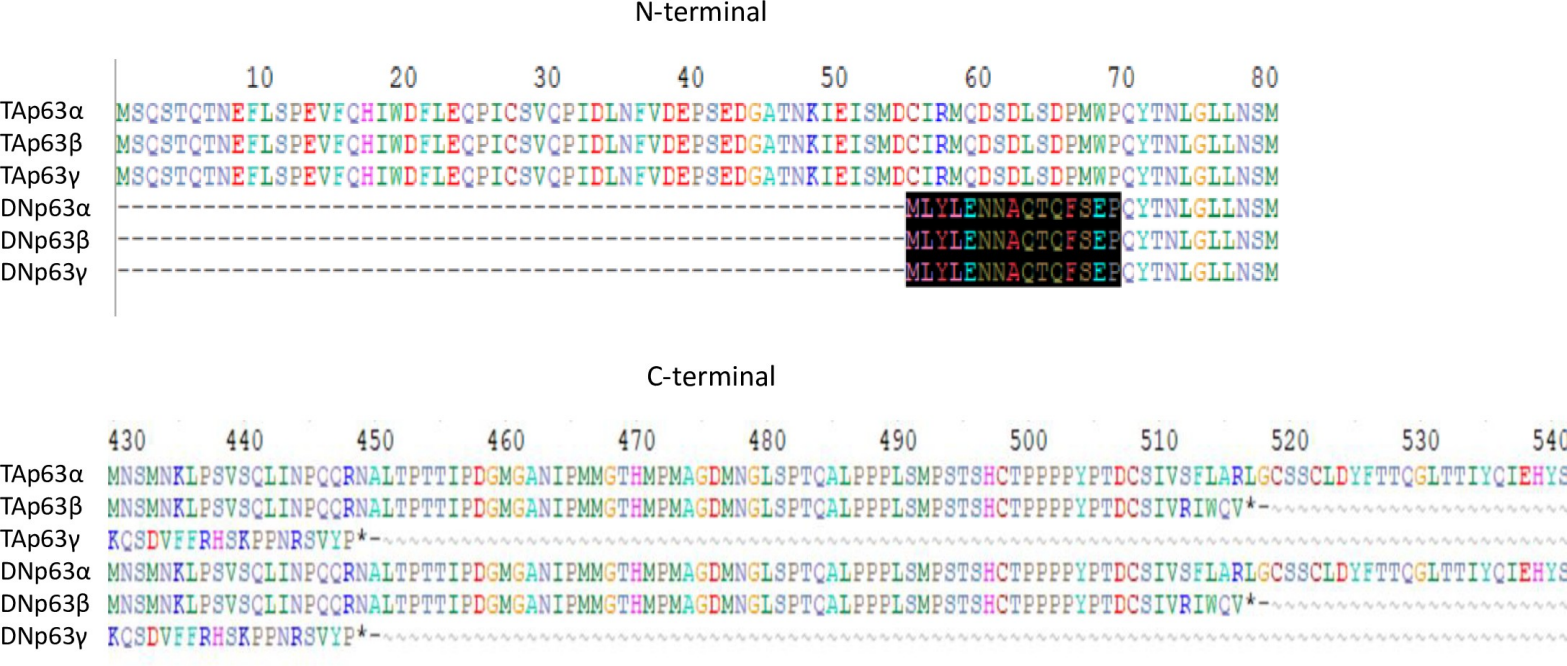
Amino acid alignment of TA and DNp63. Shaded area at N-terminal shows amino acids in DNp63 that differ from those in TAp63. C-terminal alignment reveals the differential lengths and amino acids among 3 different isoforms of p63.

Next, we investigated expression of mRNA encoding Δ*Np63* and *ABCG2* in growing cells from Lcor, Lm, and Lconj explants. P63 proteins are members of the p53 tumor suppressor protein family; this family includes TAp63, which contains transactivation (TA) domain, and ΔNp63, which lacks the TA domain. TAp63 promotes cell apoptosis, whereas ΔNp63 inhibits TAp63 and thus promotes cell survival and proliferation. TAp63 and ΔNp63 each have three isoforms, α, β, and γ, which are distinguished by differences in C-terminal sequence [24]. Among the three isoforms, the α isoform is most abundant in limbal tissue [21]. An amino acid alignment of p63 proteins is shown in Fig 3.

**Fig 4:**
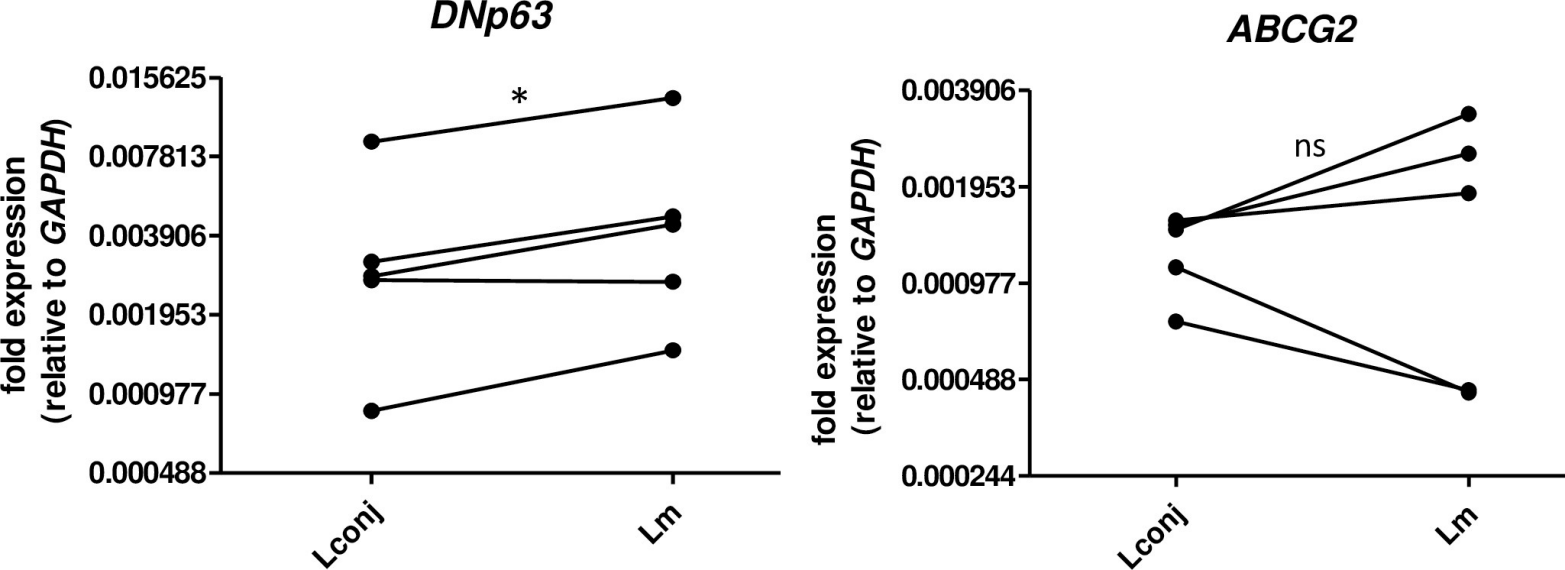
qRT-PCR detection of mRNA encoding stem cell markers Δ*Np63* and *ABCG2*. *, *p*-value = 0.02; ns, not significant.

Both Δ*Np63* and *ABCG2* are characterized markers for limbal stem cells [2, 20, 25]. As the generation of unique amplicon for Δ*Np63α* (length > 1 kb) is technically unfeasible for real-time PCR, our qRT-PCR analysis targets all three Δ*Np63* isoforms simultaneously. We compared the gene expression levels in cells from Lm and Lconj; unfortunately, the limited outgrowth obtained precluded qRT-PCR analysis of the Lcor cultures. We found that the expression of transcript encoding Δ*Np63* in cultivated cells from Lm was slightly, although significantly, higher than expression levels detected in cells from Lconj. In contrast the expression of *ABCG2* transcript (Fig 4) was not statistically different between these two sites.

**Fig 5:**
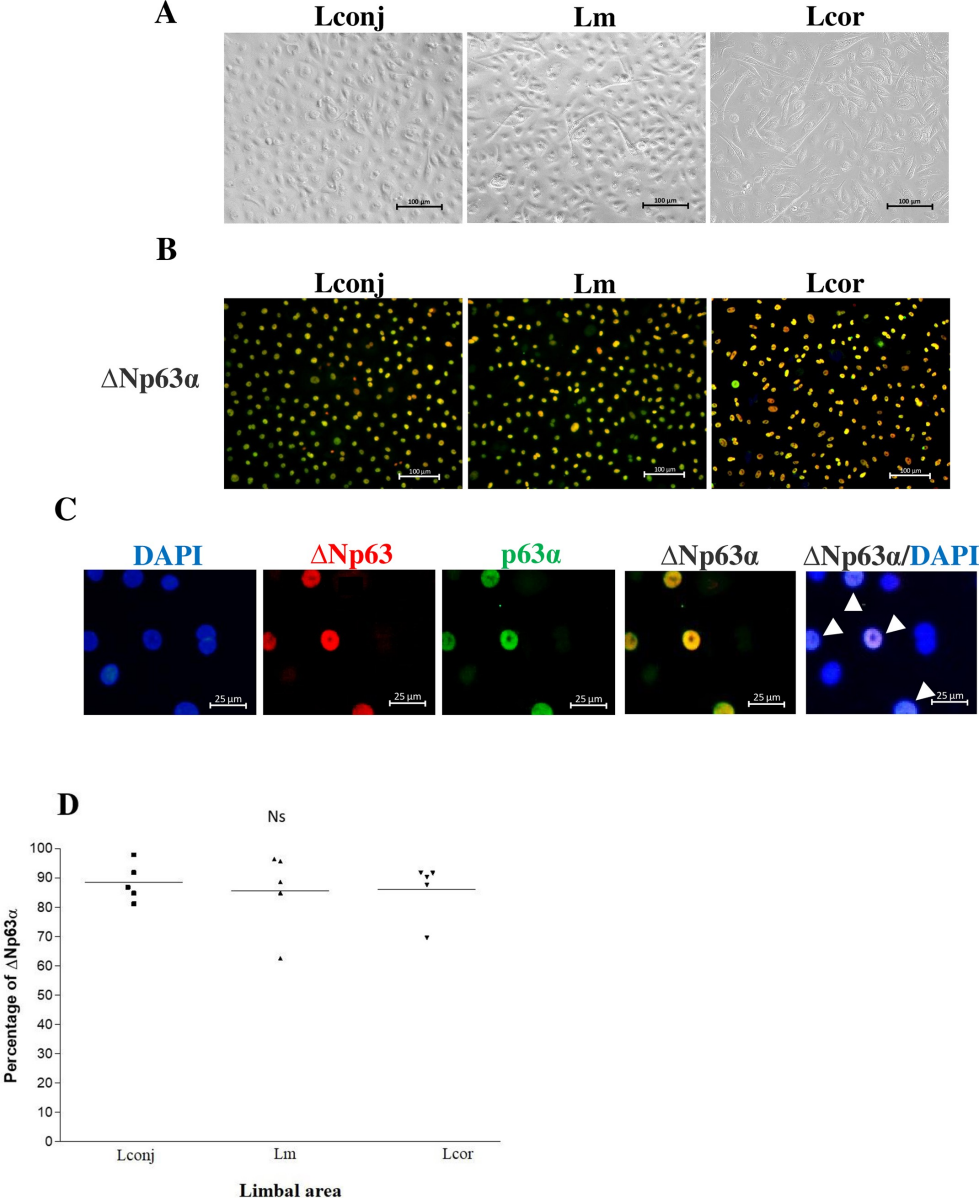
Cell morphology and detection of limbal stem cell marker, ΔNp63α. **(A)** Representative confluent cell culture and **(B)** indirect immunofluorescence detection of the cells expressing both ΔNp63 (red) and p63α (green) that represent ΔNp63α-positive limbal stem cells (yellow) in outgrowth cultures from Lconj, Lm, and Lcor (200× magnification, scale bar = 100 μm). **(C)** 200× magnification (zoom in) of the stained cells from Lm (scale bar = 25 μm); colocalization of ΔNp63 (red) and p63α (green) was detected in the nuclei (shown in yellow and white arrows). **(D)** The percent of ΔNp63α-positive cells from Lconj, Lm, and Lcor outgrowth cultures. The line represents the mean percentage of the ΔNp63α-positive cells. Ns, not significant by ANOVA for repeated measures.

Quantitative evaluation of stem cells in tissue culture was determined by immunocytochemistry (indirect immunofluorescence) staining for ΔNp63α (Fig 5). ΔNp63α is the specific stem cell marker at the basal layer of the limbus; it is also detected at high levels in the holoclone [2]. The mean percent ± standard error of mean (SEM) of ΔNp63α-positive cells in *in vitro* cultures of cells from Lconj, Lm, and Lcor was 88.40 ± 2.90, 85.51 ± 6.15, and 86.10 ± 4.23, respectively (n = 5; Fig 5).

### Localization and quantity of limbal stem cells at the three explant sites

**Fig 6:**
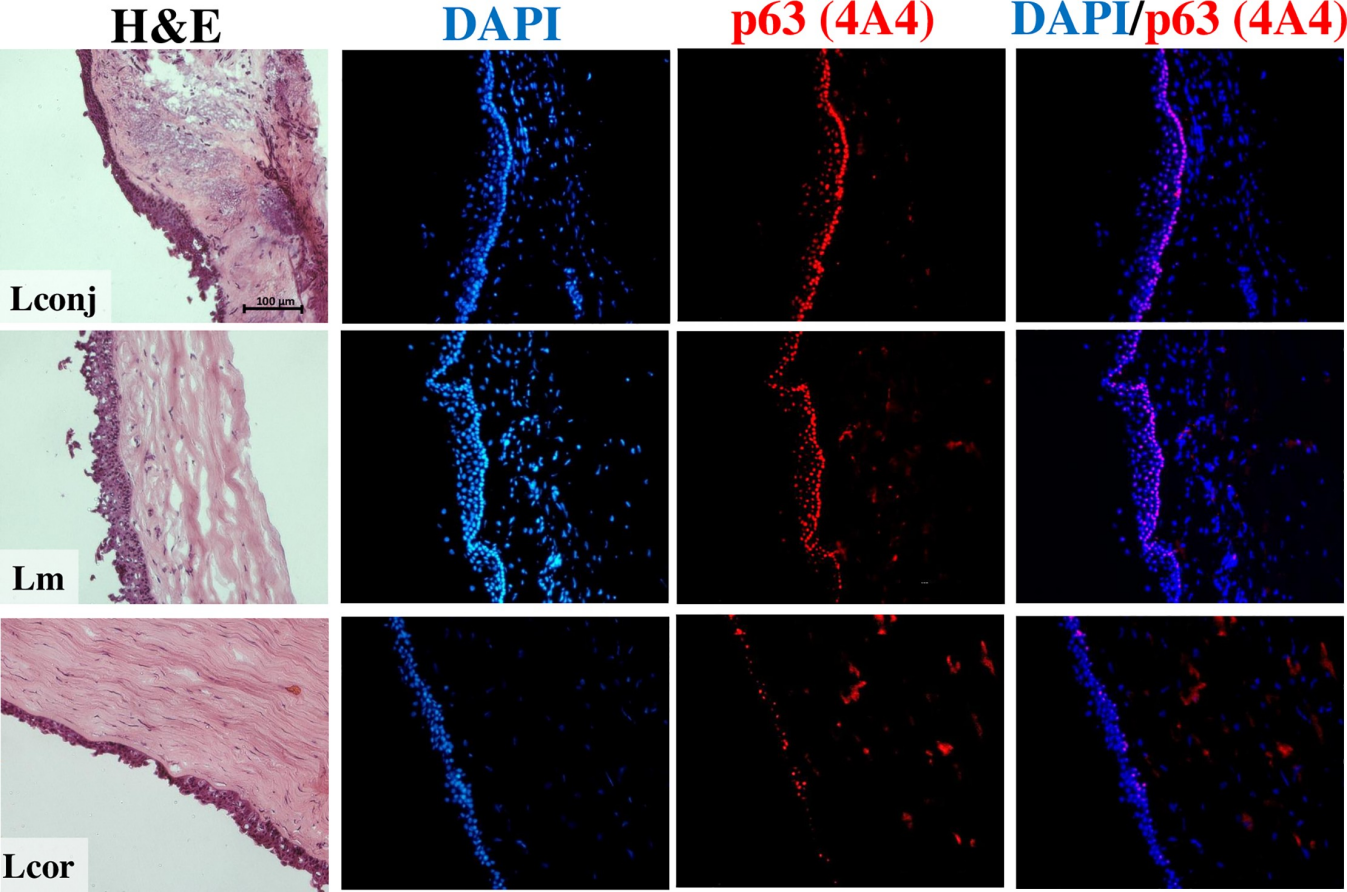
Tissue histology and detection of p63 proteins in the full-thickness limbal tissues from Lconj, Lm, and Lcor sites. Scale bar = 100 μm. Detection of p63 proteins in the basal layers of the limbus with anti-p63 Ab clone 4A4 (red) located with the nuclei (blue).

The histology of the full-thickness limbal explant was revealed by H&E staining. As shown in Fig 6, there were pronounced differences in layer and cell arrangement at the Lconj, Lm, and Lcor sites. Tissue sections from Lconj and Lm contained more prominent ridges than what was found at the Lcor site. Stem cells were located and evaluated quantitatively by indirect immunofluorescence staining with anti-p63 (4A4). This analysis revealed that p63-positive cells were more abundant within the basal layers in explants from Lconj and Lm than in those from Lcor.

## Discussion

SLET and CLET are currently used as treatments for LSCD. In CLET and SLET, a small region of the limbus is harvested for cultivation or direct transplantation, respectively. Previous studies have shown that the size of tissue explant has a significant impact on the capacity for cell proliferation. It was not at all clear whether different regions within the limbus have greater or lesser growth potential. In this study, we examined the capacity for cell proliferation and expression of stem cell markers using tissue explants from three distinct regions of the limbus, including Lcor, Lm, and Lconj. We demonstrated that Lm explants exhibited greatest capacity for proliferation *in vitro* and the highest level of expression of stem cell markers. Slightly lower levels of stem cell markers were detected in Lconj, although the cells from the explant grew well in culture. Cells from the Lcor explants did not proliferate effectively and should not be harvested for limbal stem cell transplantation. Our immunohistochemistry results revealed abundant p63-positive limbal stem cells in the basal layers of Lm and Lconj explant tissue at levels exceeding those detected in the Lcor explants. Collectively, our results suggest that limbal explants from Lm and Lconj are more suitable for CLET and SLET than those from Lcor.

Size and location of the limbus explants may have significant impact on limbal growth and proliferation *in vitro*. Utheim *et al*. reported that larger (3 mm) limbal explants yield larger outgrowths and are preferable than small (1 mm) explants, although small explant exhibited higher growth rates. No differences between small and large explants were detected when considering other parameters such as explant thickness, strength, and expression of stem cell markers [19]. The explant must be harvested from an area of the limbus that has a sufficient number of stem cells; this will permit the explanted tissue to maintain proliferative potential that is critical for a successful treatment outcome. Regarding the location of the limbus explants, cultivated limbus clones from the superior and inferior regions of the limbus were shown to have better proliferative potential than those from the nasal and temporal regions [20]. However, prior to this study, it was not clear whether proximity to the cornea or conjunctiva had an impact on stemness and differential growth potential. Our findings revealed that, overall, this region included a sizable number of stem cells and significant proliferation capacity. Furthermore, we clearly demonstrated that explants from Lconj and Lm sites yielded more stem cells and exhibited higher growth potential. These findings provide critical information for ophthalmic surgeons who currently use limbal explants for transplantation by CLET or SLET.

We also determined the expression of stem cell markers Δ*Np63* and *ABCG2* in limbal stem cell outgrowths from Lconj and Lm; the presence of these two markers has been shown to correlate with successful transplantation [2, 20, 25]. Although limbal stem cells from Lm expressed significantly higher levels of transcript encoding Δ*Np63* mRNA compared to Lconj, the difference, while statistically significant, was relatively small. This is notable because stem cells from Lconj explants grew well *in vitro* and yield large cell outgrowths. As it is sometimes difficult to distinguish between precise sites within the limbus, we suggest that both Lm and Lconj sites are acceptable areas for tissue harvesting for limbal stem cell transplantation. Although we performed qRT-PCR using a method that detects all three isoforms of Δ*Np63*, we believe that the results likely represent amplification of the α isoform, which is the most abundant isoform in limbus [21].

In addition to the expression of Δ*Np63* mRNA, we determined the number and fraction of ΔNp63α-positive stem cells in cell outgrowths from explants from three different sites by indirect immunofluorescence staining with two antibodies specific to ΔNp63 and p63α, respectively; ΔNp63α was reported as a specific marker of stem cell-derived holoclones [2]. We found that the limbal cultures from all three explants contained more than 80% ΔNp63α-positive cells. It is interesting to note that >80% of the cells in the Lcor cultures expressed ΔNp63α, but they did not proliferate effectively and are thus not suitable for transplantation. We also confirmed the expression of immunoreactive p63 in limbal tissue from cadaveric donors. While p63 is a putative limbal stem cell and transient amplifying cell (TAC) marker [26, 27], the stem cells are located within the basal layer, and TACs are detected at the supra-basal layer. We used an antibody that detects all isoforms of p63 as the antibodies that specifically detect ΔNp63 or p63α were not useful for immunohistochemistry by indirect immunofluorescence applications. However, these results most likely to represent the quantity and localization of limbal stem cells in tissue not only because of the relatively high expression of ΔNp63 but also low expression of TAp63. In addition, as noted above, ΔNp63α is the most abundant p63 isoform in limbal tissue [21]. Our finding clearly demonstrated that the basal layers of the limbal tissue at the Lconj and Lm sites are densely populated with p63-positive cells and are more abundant at these sites than in Lcor. It is not clear what the overall physiologic impact of this finding might be. We hypothesize that limbal stem cells might inhabit a niche that is associated with the anatomy of the palisades of Vogt, which are located across the limbus toward the end of conjunctival vessels [28, 29]. In addition, it may be possible that the proximity of Lconj to conjunctiva, the area with high proliferative capacity, enables Lconj to proliferate well. On the other hand, the lower proliferation capacity of Lcor is possibly because of its proximity to cornea, the area with low proliferation potential. Nonetheless, abundant expression of p63 in tissue from Lconj and Lm sites correlated well with the growth rate of limbal explants from these two sites. Collectively, these findings support our contention that explant tissue from Lm and Lconj sites are more suitable than those from Lcor sites for limbal stem cell transplantation.

Finally, our studies made use of animal-component-free CnT-Prime® culture medium for explant culture. Conventional keratinocyte growth medium contains supplements including bovine pituitary extract [30], which may not be safe for clinical use; we found that we could carry out these experiments with CnT-Prime® medium [31] that did not require supplementation with animal products. This switch was also due in large part to a shortage of keratinocyte growth medium (Lonza) that arose during our experiments. The Rho-associated, coiled-coil-containing protein kinase (ROCK) inhibitor (Y27632) has been shown to promote corneal wound healing and cell proliferation [32, 33]; we found that supplementation with Y27632 was critical for explant outgrowth in this medium. The potential clinical benefits of Y27632 should be evaluated further; there might be a role for this inhibitor as a supplement in eye drops for postoperative treatment in patients undergoing SLET.

In conclusion, we present a culture system that promotes stem cell proliferation from limbal explant tissue *in vitro*. The fraction of ΔNp63α-positive cells detected among cell outgrowths in these cultures indicates that this method supports and maintains the stemness. Furthermore, our culture conditions are serum-, animal product-, and feeder-free and are thus suitable for clinical application. Furthermore, we identified optimal sites for limbal explants that may result in improved cultivation and higher rates of successful transplantation. Specifically, we have shown that isolation of tissue from the middle limbus (Lm) or from sites immediately adjacent to the conjunctiva (Lconj) will likely lead to successful outgrowth and cultivation. Collectively, our findings may help ophthalmic surgeon toward successful treatment outcomes when using CLET and SLET in patients with LSCD.

## Supporting information

S1 Fig Growth of limbal stem cells from explants in CNT-Prime media with or without a ROCK inhibitor Y-27632. (PPTX)
