# Supplementary material for: Characterization of limbal explant sites: Optimization of stem cell outgrowth in in vitro culture
Source: PLoS One. 2020 May 14;15(5):e0233075. doi: 10.1371/journal.pone.0233075 (PMC7224544; doi:10.1371/journal.pone.0233075)
Supplement: S1 Fig — (PPTX) [file pone.0233075.s001.pptx]

## Slide 1
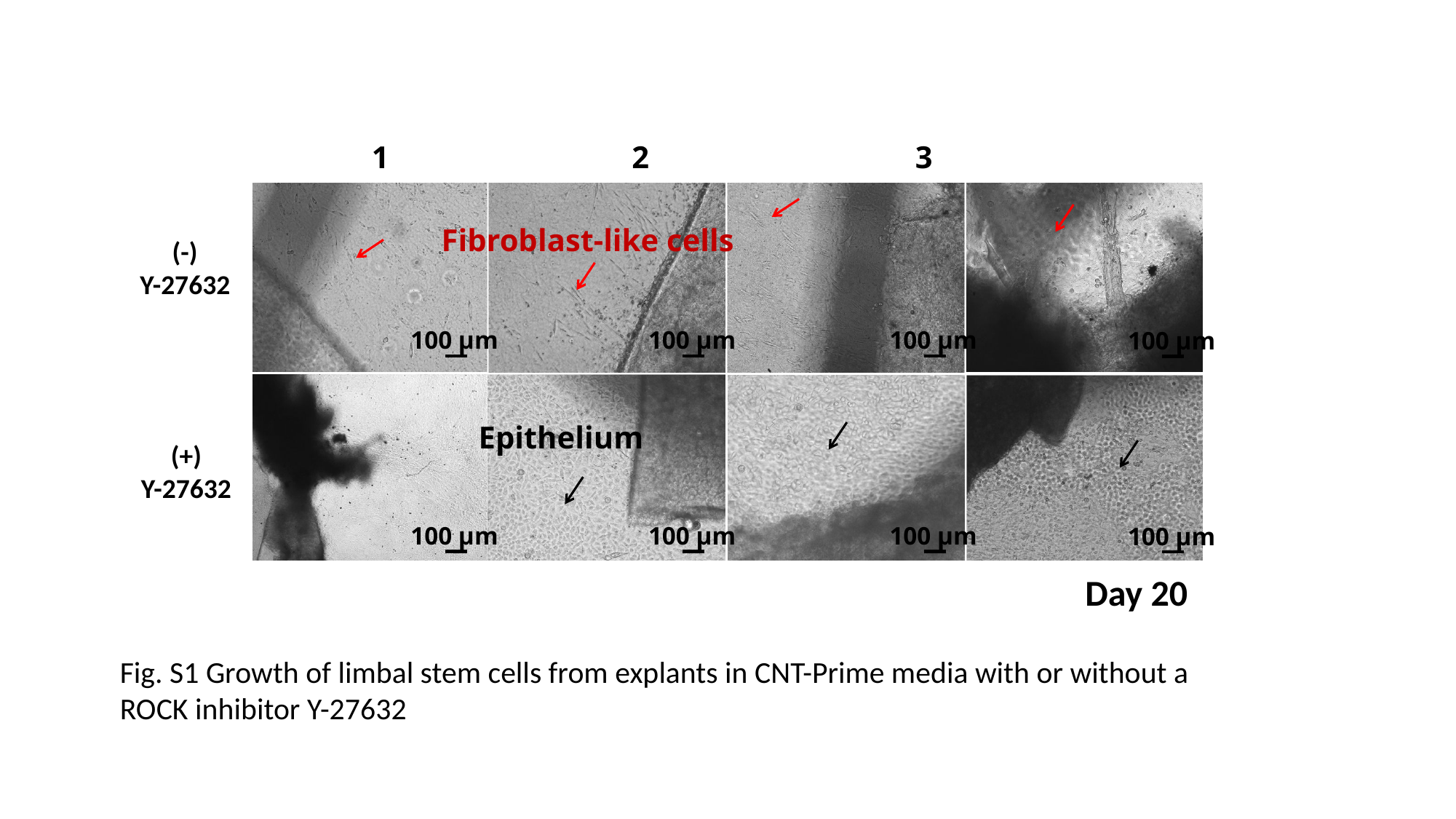

1 2 3 4
Fibroblast-like cells
(-)
Y-27632
100 µm
100 µm
100 µm
100 µm
Epithelium
(+)
Y-27632
100 µm
100 µm
100 µm
100 µm
Day 20
Fig. S1 Growth of limbal stem cells from explants in CNT-Prime media with or without a ROCK inhibitor Y-27632
